# Mechanistic interactions of diet, exercise, and pharmacotherapy in skeletal muscle metabolism: evidence and translational perspectives

**DOI:** 10.3389/fphys.2026.1957316

**Published:** 2026-09-22

**Authors:** Yan Wang, Mengran Qin, Xuezhi Liu, Jianxiong Ma

**Affiliations:** 1 Tianjin Hospital, Tianjin University, Tianjin, China; 2 Tianjin Orthopedic Institute, Tianjin, China; 3 Tianjin Key Laboratory of Orthopedic Biomechanics and Medical Engineering, Tianjin, China

**Keywords:** dietary intervention, exercise, mechanism integration, pharmacotherapy, skeletal muscle metabolism

## Abstract

Skeletal muscle plays a central role in whole-body energy homeostasis, metabolic flexibility, and insulin sensitivity, acting as a key integrator of nutritional, mechanical, and pharmacological signals. Although diet, exercise, and pharmacotherapy are often investigated as separate interventions, they converge on shared regulatory pathways in skeletal muscle, including nutrient sensing, mechanotransduction, mitochondrial quality control, inflammatory remodeling, and anabolic–catabolic balance. Through core signaling networks such as AMPK–mTOR, SIRT1/PGC-1α, and insulin/AKT pathways, these interventions may interact in additive, complementary, synergistic, or antagonistic manners depending on physiological status, disease context, baseline metabolic condition, and intervention timing. This Review critically integrates current evidence on how diet, exercise, and pharmacotherapy regulate skeletal muscle metabolism across aging, metabolic disease, and adaptive physiological states. We propose a muscle-centered integrative framework organized around five major regulatory domains: substrate utilization and metabolic flexibility, mitochondrial remodeling, protein turnover, inflammatory control, and regenerative capacity. Within this framework, we highlight how intervention responses are shaped by key biological and clinical modulators, including age, disease state, muscle fiber composition, training status, nutritional background, and baseline metabolic health. Importantly, we distinguish findings supported by human studies from those derived primarily from preclinical or mechanistic models, thereby emphasizing differences in translational reliability. Finally, we discuss current limitations in interpreting combined interventions and outline future directions for precision-oriented strategies that integrate nutrition, exercise, and pharmacotherapy to optimize skeletal muscle function and systemic metabolic health.

## 1 Introduction

Skeletal muscle is one of the largest and most metabolically active tissues in the human body, accounting for approximately 40% of total body mass (Frontera and Ochala, 2015). It plays an irreplaceable role in maintaining motor function and energy homeostasis. Beyond fundamental physiological functions like mechanical support and movement execution, skeletal muscle plays a central role in systemic glucose uptake, fatty acid oxidation, and amino acid metabolism (Lin et al., 2026). Its metabolic state plays an important role in whole-body energy homeostasis and metabolic flexibility (Lin et al., 2026). Extensive research indicates that impaired skeletal muscle metabolism is frequently accompanied by pathological alterations such as insulin resistance, mitochondrial dysfunction, and reduced energy conversion efficiency (Hesselink et al., 2016; Lin et al., 2026). These abnormalities are recognized as a critical common foundation for multiple chronic metabolic disorders, including obesity, type 2 diabetes, sarcopenia, and metabolic syndrome (Pedersen and Febbraio, 2012; Hesselink et al., 2016).

Among the numerous factors regulating skeletal muscle metabolic homeostasis, diet, exercise, and pharmacotherapy have garnered significant attention due to their high controllability, well-defined molecular targets, and promising clinical translation potential (Kanie et al., 2020). Dietary factors not only provide essential nutritional substrates for skeletal muscle protein synthesis and energy metabolism but also regulate myoprotein synthesis rates, substrate selection, and metabolic adaptive remodeling through multiple nutrient-sensing pathways. Research indicates that prolonged low-protein intake or insufficient energy supply is closely associated with skeletal muscle mass decline, reduced muscle strength, and the onset of sarcopenia (Cao et al., 2022), suggesting the fundamental role of a balanced dietary structure in maintaining skeletal muscle metabolic function. In contrast, exercise intervention, particularly endurance training, is recognized as a key exogenous stimulus for inducing skeletal muscle metabolic reprogramming. It promotes mitochondrial biogenesis, enhances lipid oxidation capacity, and improves metabolic adaptability by activating energy-sensing and mitochondrial regulatory pathways such as AMPK and PGC-1α (Mader et al., 2022).

Beyond lifestyle interventions, pharmacotherapy remains crucial in the prevention and treatment of metabolic diseases. Various metabolic drugs can restore skeletal muscle metabolic homeostasis to some extent by improving insulin signaling, regulating energy metabolism pathways, and suppressing chronic low-grade inflammation (55 EASD annual meeting of the European association for the study of diabete: Barcelona, Spain, 16-20 September 2019, 2019). For instance, the antidiabetic agent Astragalus polysaccharide has been reported to significantly enhance skeletal muscle insulin sensitivity and improve systemic glucose homeostasis, further highlighting the potential value of pharmacotherapy in regulating skeletal muscle metabolism (Liu et al., 2013). However, existing research predominantly focuses on single intervention models. Systematic integration of knowledge regarding the interactions between different intervention methods under multiple conditions and their potential interaction mechanisms remains lacking. Furthermore, the impact of individual metabolic background differences on intervention responses requires further elucidation.

Against this background, an important unresolved question is not simply how diet, exercise, or pharmacotherapy independently influence skeletal muscle metabolism, but how these interventions converge, interact, and sometimes diverge within shared regulatory networks. Rather than viewing them as isolated strategies, a muscle-centered perspective may provide a more informative framework for understanding combined metabolic adaptation and therapeutic response. In this Review, we integrate current evidence across nutritional, exercise, and pharmacological studies and organize it around five common domains of skeletal muscle regulation: substrate utilization, mitochondrial quality control, protein turnover, inflammatory remodeling, and regenerative capacity. We further assess the strength and limitations of the available evidence, distinguish well-supported human findings from predominantly preclinical observations, and discuss the translational implications of combining interventions across aging, metabolic disease, and rehabilitation settings. By doing so, we aim to move the field from parallel description of intervention effects toward a more mechanism-based framework for precision metabolic intervention. To clarify this integrative perspective, the conceptual framework of this Review is summarized in **Figure 1**.

**Figure 1:**
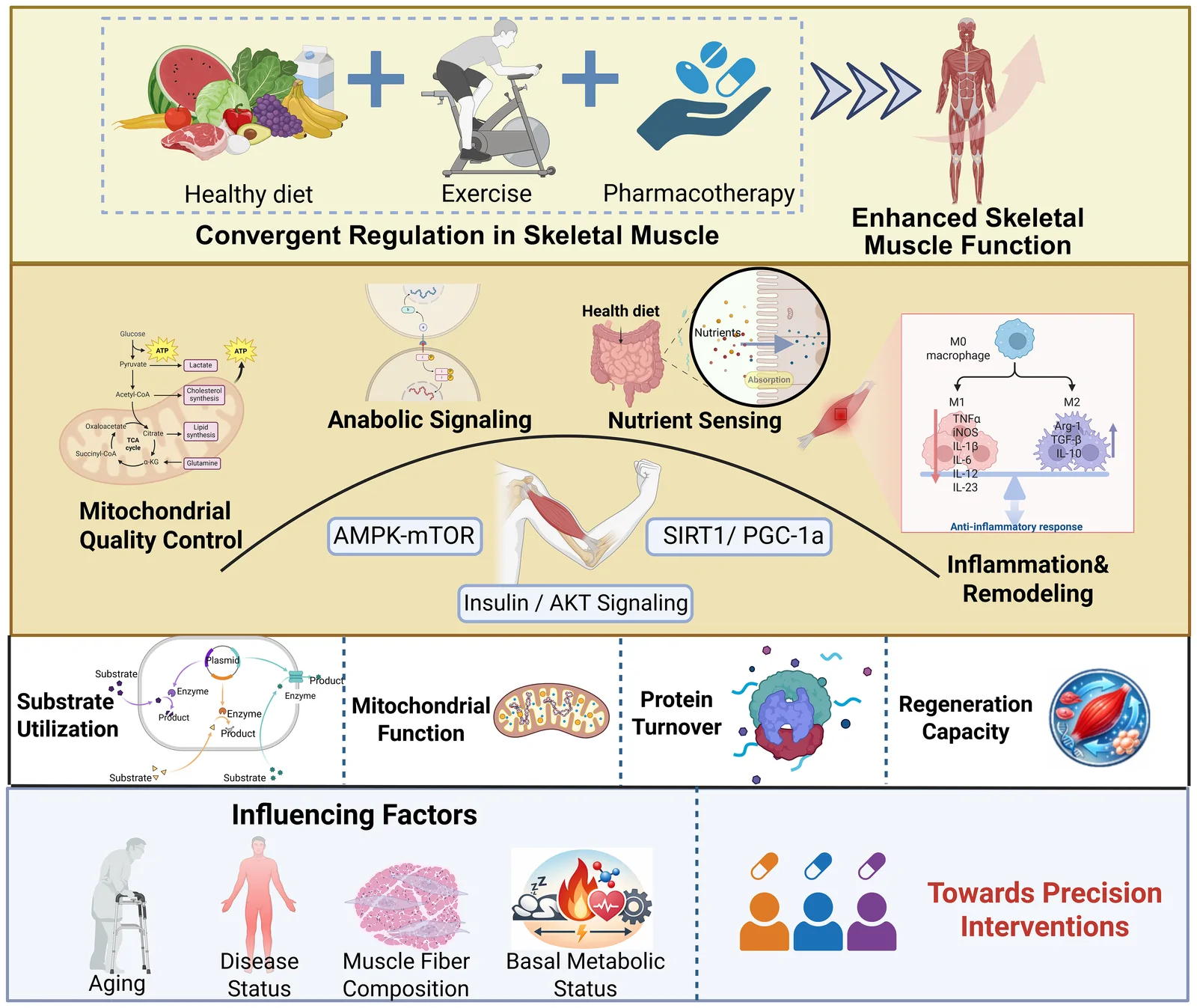
Skeletal muscle as a convergence node integrating dietary, exercise, and pharmacotherapy. Diet, exercise, and pharmacotherapy regulate skeletal muscle through partially overlapping rather than fully independent mechanisms. Their effects converge on shared regulatory domains, including nutrient sensing and anabolic signaling, substrate utilization and metabolic flexibility, mitochondrial remodeling and quality control, inflammatory remodeling, protein turnover, and regenerative capacity. These integrated responses are coordinated through key signaling pathways such as AMPK-mTOR, SIRT1/PGC-1α, and insulin/AKT, but their overall direction and magnitude are further shaped by biological context, including aging, disease status, muscle fiber composition, and baseline metabolic condition. Through this muscle-centered integrative network, multimodal interventions collectively influence muscle function, insulin sensitivity, and whole-body metabolic health. Created in BioRender. Liu, X. (2026). https://BioRender.com/08z7yqz.

To support this narrative Review, a structured search of PubMed/MEDLINE, Web of Science, and Scopus was conducted from database inception through June 2026 using terms related to skeletal muscle metabolism, diet/nutrition, exercise, pharmacotherapy, and major metabolic pathways. Studies relevant to skeletal muscle regulation, shared mechanisms, or combined interventions were prioritized; less relevant studies were not emphasized. Human interventional evidence was prioritized, with observational, animal, and *in vitro* studies used when mechanistically informative or when human evidence was limited. Evidence was qualitatively appraised by study design, consistency, directness to skeletal muscle outcomes, and availability of human evidence. Negative or conflicting findings were considered, and mechanistic convergence was not regarded as synergy without direct combined-intervention studies or interaction analyses.

## 2 Dietary protein, amino acid availability, and nutrient sensing in skeletal muscle metabolism

Skeletal muscle protein metabolism is highly sensitive to nutritional status, and dietary protein and its amino acid composition are considered key nutritional factors for maintaining skeletal muscle structural integrity and metabolic function (Deane et al., 2021). Contrary to the traditional view that proteins serve solely as substrates for synthesis, growing evidence indicates dietary proteins systematically engage in skeletal muscle protein synthesis, degradation, and metabolic remodeling through amino acid sensing systems, energy state regulation, and multi-level signaling network integration (Bond, 2016). This comprehensive involvement shapes the metabolic phenotype of skeletal muscle during physiological adaptation, aging, and disease states (Argilés et al., 2016).

In the context of metabolic stress or disease, dietary protein may exert a particularly prominent influence on skeletal muscle amino acid metabolism. For example, in a rat model with reduced kidney mass, skeletal muscle showed significantly increased release of alanine, tyrosine, and phenylalanine in skeletal muscle. These changes were primarily regulated by dietary protein levels and insulin sensitivity, with glucose uptake not acting as a limiting factor. This suggests that protein intake plays a relatively independent role in regulating muscle amino acid metabolism (Harter et al., 1979). Population studies reveal that post-bariatric surgery in individuals over 65 years old, despite overall fat-free mass reduction, showed no significant decrease in standardized muscle mass compared to non-surgical cohorts. These changes were primarily influenced by age, obesity severity, and protein intake levels, with low protein intake closely associated with sarcopenia risk (Eksteen et al., 2025). Additionally, under identical protein intake conditions, low-carbohydrate diets may further exacerbate denervation-induced skeletal muscle atrophy, suggesting significant interactions between macronutrient ratios and protein metabolism (Yokogawa et al., 2025). Beyond intake levels, protein quality and amino acid composition profoundly influence skeletal muscle metabolism. Low-quality protein intake may compromise muscle and bone health (Aggarwal and Bains, 2022). However, protein quality cannot be characterized solely by grain dominance or lysine content, because the limiting indispensable amino acid varies across protein sources; lysine is often limiting in cereals, whereas methionine and cysteine are more frequently limiting in legumes (Hertzler et al., 2020). At the population level, however, some studies indicate that in middle-aged and older adults, dietary protein intake shows no significant association with muscle strength decline, suggesting its effects may be modulated by physical activity levels or individual health status (Kim et al., 2021). Accordingly, combining complementary protein sources may provide a more balanced indispensable amino acid profile (van Vliet et al., 2015). Essential amino acid availability is particularly important for stimulating muscle protein anabolism, as demonstrated in older adults (Volpi et al., 2003).

At the molecular mechanism level, branched-chain amino acids (BCAAs) and their metabolic pathways occupy a central position in skeletal muscle metabolic regulation. Impaired BCAA catabolism is considered one of the key molecular foundations of sarcopenia, directly impacting muscle mass and function (Zuo et al., 2025). In critically ill patients, despite relatively preserved protein digestion and amino acid absorption capacity, skeletal muscle protein synthesis is significantly constrained, exhibiting a classic “anabolic resistance” (Chapple et al., 2022). In older adults, combining exercise with essential amino acid provision may help overcome anabolic resistance by enhancing mTORC1 signaling and muscle protein synthesis (Horwath et al., 2025), whereas in prolonged critical illness the anabolic response to amino acid supplementation remains less predictable (Chapple et al., 2022; Wittholz et al., 2024). Conversely, during exercise recovery, dietary protein intake exhibits a pronounced dose-dependent effect on muscle anabolism. The magnitude and duration of amino acid incorporation into muscle proteins increase with higher intake levels, with no clear upper limit observed to date (Trommelen et al., 2023). In disease-related muscle wasting scenarios, amino acid metabolism disorders and energy imbalances further exacerbate skeletal muscle damage. Cancer cachexia can lead to mitochondrial dysfunction, cationic amino acid metabolism imbalance, and decreased energy charge, thereby accelerating protein degradation and muscle atrophy (Kunzke et al., 2020). In advanced cancer patients, elevated myofibrillar protein breakdown and amino acid turnover rates are closely associated with muscle weakness and functional decline (van der Meij et al., 2019). Supplementing essential amino acids during the perioperative period or under specific conditions can mitigate postoperative muscle wasting and enhance protein synthesis (Ueyama et al., 2020), though its efficacy in prolonged critical states remains uncertain (Wittholz et al., 2024).

At the level of healthy aging and metabolic adaptation, amino acid sensing and signal transduction are equally critical determinants of skeletal muscle metabolic plasticity. In lean and healthy elderly males, exercise combined with essential amino acid intake significantly enhances muscle protein synthesis rates and mTORC1 signaling, with their anabolic sensitivity closely correlated to elevated expression of amino acid sensors and related proteins (Horwath et al., 2025). Furthermore, dietary essential amino acid balance plays a vital role in diet-restriction-induced lifespan extension, renal protection, and muscle function maintenance (Yoshida et al., 2018). Long-term protein restriction mediates skeletal muscle metabolic remodeling by regulating muscle amino acid composition, transporter expression, and mTOR signaling pathways (Yin et al., 2017). It is noteworthy that dietary protein significantly influences skeletal muscle metabolism with considerable individual variation. The distribution of muscle fiber types in individuals with differing insulin sensitivities is closely associated with specific amino acid metabolic characteristics (Magnusson et al., 2025). Amino acids and their metabolites (such as leucine and β-hydroxy-β-methylbutyrate) not only serve as substrates for synthesis or energy but also directly participate in regulating muscle function (Kamei et al., 2020). At the population level, higher intake of BCAAs is independently associated with superior skeletal muscle mass indices (Chae et al., 2020). Regarding protein sources, pea protein and whey protein induce similar amino acid responses and maintain myosin protein synthesis across different age groups (Salles et al., 2024). Evidence from highly trained populations provides a physiological example of how protein and essential amino acid availability interact with training-induced adaptation (Baranauskas et al., 2023; Jeong et al., 2024). Among athletes, resistance training combined with essential amino acid intake significantly promotes muscle mass gain and improves inflammatory status (Jeong et al., 2024). Protein and essential amino acid intake levels in elite athletes are also closely associated with training-induced muscle adaptation (Baranauskas et al., 2023). Overall, dietary protein and its amino acid composition systematically regulate skeletal muscle metabolic remodeling through multiple signaling networks and metabolic pathways. Future research should further elucidate their interactions with exercise and pharmacological interventions under diverse physiological and pathological conditions, providing theoretical foundations for precise, personalized nutritional intervention strategies.

## 3 Effects and mechanisms of exercise on skeletal muscle metabolism

Exercise is one of the most important and highly plastic non-pharmacological interventions for regulating skeletal muscle metabolic homeostasis. Its metabolic effects extend beyond enhancing muscle mass or exercise capacity, instead systematically reshaping the structural foundation, energy metabolism patterns, and regenerative potential of skeletal muscle through multi-level mechanisms. Extensive research demonstrates that exercise of varying types, intensities, and training patterns can restore or enhance skeletal muscle metabolic function across diverse disease states, aging, and metabolic disorders. This occurs by improving microcirculatory perfusion, regulating protein synthesis and degradation balance, activating energy-sensing and growth signaling pathways, and reshaping metabolism-related transcriptional and epigenetic programs. These adaptive changes not only explain exercise’s protective role in metabolic and chronic wasting diseases but also provide a crucial biological basis for its combined application with dietary and pharmacological interventions.

Because skeletal muscle responses are protocol-dependent, the evidence below is interpreted according to exercise modality and intensity where reported, while recognizing that exercise duration and frequency vary substantially across the cited studies and cannot be uniformly classified across all mechanistic outcomes.

At the level of tissue structure and metabolic signaling, exercise first lays the foundation for metabolic remodeling by improving skeletal muscle microcirculation and oxygen delivery capacity. Disease-specific studies further illustrate that the vascular mechanisms engaged by exercise may differ according to the underlying pathology (Spee et al., 2016; Merlet et al., 2019). Moderate-intensity endurance training significantly increases skeletal muscle capillary density and exchange area in sickle cell disease patients, partially reversing microvascular remodeling defects and improving exercise capacity (Merlet et al., 2019). In contrast, high-intensity interval training primarily acts in chronic heart failure patients by optimizing skeletal muscle microvascular oxygen supply-demand matching and accelerating oxygen uptake kinetics, rather than relying on increased cardiac output (Spee et al., 2016). Under pathological conditions dominated by inflammation and catabolism, moderate-intensity therapeutic exercise alleviates muscle atrophy and improves strength by inhibiting MuRF1-mediated protein degradation and reducing G-CSF levels, thereby limiting neutrophil infiltration. This highlights exercise’s regulatory capacity over the metabolic-inflammatory axis (Files et al., 2015). Both resistance and endurance exercise activate the Akt/FoxO pathway to suppress chronic kidney disease-induced protein degradation. Resistance exercise further enhances protein synthesis and improves myogenic progenitor cell function via the mTOR–p70S6K axis, demonstrating the metabolic regulation specificity of different exercise modalities (Wang et al., 2009). Blood flow restriction training effectively induces strength gains and mitigates muscle atrophy under low-intensity loads, offering a safe metabolic intervention strategy for frail or bedridden individuals (Conceição and Ugrinowitsch, 2019). Concurrently, exercise influences hypertrophy, metabolism, and insulin sensitivity across different muscle fiber types through IGF-1/PI3K/Akt signaling, Caveolin-1 regulation, extracellular matrix (ECM) remodeling, and growth factor expression changes (Oh et al., 2007; Gumpenberger et al., 2020; Feng et al., 2022). Individual variations in response to training stimuli are determined by muscle fiber type composition, capillarization levels, and baseline metabolic status (Snijders et al., 2017; Kovac et al., 2024).

Mitochondrial functional remodeling serves as the pivotal mechanism through which exercise enhances skeletal muscle metabolic capacity. Exercise training activates signaling pathways including PGC-1α, AMPK/Sirt1, and Nrf2-Drp1 to coordinate mitochondrial biogenesis, dynamic changes, and quality control. This process elevates fatty acid and branched-chain amino acid oxidation capacity while improving insulin sensitivity (Overmyer et al., 2015; Mulya et al., 2017; Yan et al., 2022). Multiple studies indicate that exercise-induced mitochondrial adaptation involves not only quantitative changes but also volumetric expansion, regulation of protein acetylation status, and optimization of electron transport chain function. Citrate synthase activity serves as a key marker of this adaptive process (Meinild Lundby et al., 2018; Tsuda et al., 2018). In various disease models, exercise restores mitochondrial dysfunction caused by high-fat diets, Parkinson’s disease, 5-aminoimidazole-4-carboxamide ribonucleotide formyltransferase/IMP cyclohydrolase (ATIC) deficiency, and chronic kidney disease, reduces reactive oxygen species (ROS)-related damage, and improves exercise tolerance (Nishikawa et al., 2015; Jun et al., 2024; Tung et al., 2024; Peng et al., 2025). However, in pathological states such as chronic obstructive pulmonary disease (COPD) or hypertension, exercise still exhibits limitations in improving mitochondrial biogenesis and antioxidant defenses, suggesting that the disease itself may suppress the plasticity of mitochondrial regulatory networks (Fiorenza et al., 2019; Abdellaoui et al., 2025). Furthermore, exercise-mediated regulation of ROS production exhibits fiber-type and substrate-selective dependencies, achieving fine-tuned control over oxidative stress through UCP3 upregulation, altered membrane composition, and enhanced endothelial antioxidant capacity (Molnar et al., 2006; Jiang et al., 2009; Hansen et al., 2022).

Beyond metabolic regulation, exercise further consolidates its long-term metabolic effects by activating skeletal muscle regeneration programs. Exercise promotes myofibrillar repair and regeneration through satellite cell activation, myonucleus migration, and local mRNA delivery (Roman et al., 2021), while maintaining mitochondrial metabolism and proliferative capacity in myoblasts by inhibiting TGF-β signaling, restoring Cyclin D1 expression, and regulating the Igfbp7-Akt-mTOR axis (Brett et al., 2020; Chen et al., 2020). Endurance exercise also promotes an increase in slow-twitch fiber proportion and enhances metabolic endurance through the SPHK1-S1P-S1PR axis, Ythdf1-myostatin pathway, and nNOS-mediated angiogenesis (Baum et al., 2023; Huang et al., 2025; Leng et al., 2025). In an aging context, lifelong exercise systematically improves skeletal muscle regenerative capacity and metabolic homeostasis by reducing proinflammatory factors, activating autophagy, and enhancing BDNF/AKT signaling (Gao et al., 2020). Overall, exercise constructs a highly dynamic and plastic metabolic regulatory network by coordinating skeletal muscle structural adaptation, metabolic signaling transduction, mitochondrial quality control, and regenerative mechanisms. Its effects are jointly influenced by exercise patterns, individual physiological states, and disease contexts. This multi-level regulatory framework provides crucial theoretical support for understanding the integrated metabolic effects exercise combined with dietary and pharmacological interventions, while also laying a solid foundation for developing precision metabolic intervention strategies.

## 4 Molecular regulation of skeletal muscle metabolism by pharmacotherapy

Skeletal muscle is not only a passive target organ affected by metabolic disorders but also a crucial site where various drugs exert systemic metabolic effects. A growing body of research indicates that different types of metabolic drugs, hormonal preparations, and novel small-molecule compounds can reshape muscle metabolic phenotypes by directly or indirectly regulating skeletal muscle glucose, lipid, and amino acid metabolic pathways, thereby influencing systemic insulin sensitivity and energy homeostasis. In this process, skeletal muscle’s capacity to integrate energy-sensing signals, inflammatory states, and hormonal stimuli determines the intensity and individual variability of drug-induced metabolic effects. The myogenic factor MG53 induces skeletal muscle insulin resistance by inhibiting insulin receptor signaling, and its neutralization significantly improves systemic metabolic homeostasis (Wu et al., 2019), suggesting that targeting myogenic signaling itself can yield systemic metabolic benefits.

Among classic hypoglycemic and metabolic regulatory drugs, the AMPK-PI3K-AKT-GLUT4 axis constitutes the core signaling network for skeletal muscle drug response. Pharmacological activation of skeletal muscle AMPK significantly enhances glucose uptake and improves systemic glucose metabolism, making it a key therapeutic target for anti-diabetic treatment (Cokorinos et al., 2017). Conversely, AMPK-mediated phosphorylation of ACC2 is crucial for maintaining fatty acid oxidation and insulin sensitivity; its functional deficiency leads to lipid accumulation and insulin resistance (O'Neill et al., 2014). Multiple drugs and bioactive compounds, including metformin, acacetin, benzothiazole derivatives, and thiazolidinedione-triazole hybrids, promote GLUT4 translocation and enhance skeletal muscle glucose uptake by activating either the AMPK or PI3K pathway (Meltzer-Mats et al., 2013; Kwon et al., 2020; Kushawaha et al., 2025). Notably, metformin not only exerts its classic AMPK-dependent effects but also promotes FNDC5/irisin release through AMPK-independent pathways or inhibits lncRNA Dreh to enhance skeletal muscle glucose transport, demonstrating its multi-level role in muscle metabolic regulation (Li et al., 2015; Takahashi et al., 2019). Furthermore, the Baf60c–Deptor–AKT pathway enables skeletal muscle to directly sense glucose and regulate postprandial blood glucose homeostasis. This pathway also contributes to the hypoglycemic effects of sulfonylurea drugs (Meng et al., 2017), while thiazolidinediones alleviate lipotoxicity-associated insulin resistance by improving skeletal muscle lipid homeostasis and oxidative capacity (Ide et al., 2000; Wessels et al., 2015). In contrast, acute rosiglitazone treatment in healthy individuals did not significantly affect muscle glucose transport, suggesting its metabolic effects may depend on long-term exposure or inter-tissue interaction mechanisms (Skrobuk et al., 2010).

Beyond traditional metabolic drugs, hormones, inflammatory modulators, and cardiovascular-related medications also profoundly influence skeletal muscle metabolic states. The β_2_-adrenergic receptor agonist clenbuterol induces skeletal muscle metabolic reprogramming and enhances glucose utilization (Meister et al., 2022), while angiotensin II impairs insulin-mediated glucose uptake by inhibiting muscle microvascular dilation, revealing the role of vascular dysfunction in muscle insulin resistance development (Premilovac et al., 2019). Glucagon-like peptide-1 (GLP-1) can induce substantial weight loss, but reductions in lean and skeletal muscle mass may accompany this response, raising concerns regarding muscle preservation, particularly in older adults (Pantazopoulos et al., 2025; Simsek and Ucar, 2026). Clinical evidence primarily concerns body-composition effects, whereas evidence for direct muscle-preserving mechanisms remains largely preclinical. In experimental models, the long-acting GLP-1 analog liraglutide promotes myogenesis and improves muscle atrophy by activating muscle GLP-1 receptors and inhibiting MuRF1 and MAFbx expression (Gurjar et al., 2020; Fan et al., 2023; Ceasovschih et al., 2025), while combined activation of GLP-1 receptors with Activin II receptor blockade further preserves skeletal muscle mass and enhances metabolic benefits (Nunn et al., 2024). The effects of glucocorticoids on skeletal muscle metabolism exhibit high context dependence. They can mediate muscle atrophy via Klf9 (Zhang Y. et al., 2025), influence mitochondrial function through non-genomic mechanisms or circadian rhythm-dependent PGC1α pathways under specific conditions (Passaquin et al., 1998; Quattrocelli et al., 2022), and demonstrate significant sex dimorphism (Salamone et al., 2022). Furthermore, estrogen, androgen, thyroid hormone, and growth hormone can directly regulate skeletal muscle energy metabolism, fiber type, and regenerative potential through their respective receptors, but their metabolic effects show marked variations across different populations (Enns et al., 2008; Mak et al., 2022; Ghaibour et al., 2023; Netzer et al., 2023; Johnston et al., 2025).

In recent years, novel targets for muscle atrophy and cachexia have continuously emerged, further expanding the conceptual boundaries of skeletal muscle as a core organ for drug intervention. TAOK1, the SIRT1-NOX4 axis, and GDF8/Activin A signaling have been demonstrated to play crucial roles in cancer cachexia-associated muscle protein degradation. Their targeted inhibition significantly alleviates muscle wasting and improves mitochondrial function (Dasgupta et al., 2020; Gonzalez Trotter et al., 2025; Zhang R. et al., 2025). Concurrently, enhancing mitochondrial homeostasis by elevating NAD levels or activating SIRT1/PGC-1α signaling has become a crucial strategy for restoring skeletal muscle metabolic capacity (Hulmi et al., 2020; Song et al., 2025). In summary, pharmacotherapy acts on skeletal muscle metabolic networks through multiple targets and pathways. Their metabolic effects depend not only on the activation or inhibition of individual signaling pathways but also heavily rely on the integrated regulation of dietary background, exercise status, hormone levels, and individual metabolic characteristics. In the future, treating skeletal muscle as a core node integrating nutritional, exercise, and drug signals holds promise for providing new theoretical foundations and practical pathways for precision metabolic interventions and personalized treatments for muscle-related metabolic diseases.

## 5 Interactions among diet, exercise, and pharmacotherapy in skeletal muscle and their clinical implications

At the level of skeletal muscle, mechanistic convergence among diet, exercise, and pharmacotherapy does not necessarily imply additive or synergistic effects. These interventions often act through a limited number of shared processes, including nutrient sensing, substrate selection, mitochondrial remodeling, redox signaling, and protein turnover (Lin et al., 2026). Whether the final outcome is beneficial therefore depends on whether the combined signals reinforce the same adaptive program, act on complementary pathways, or interfere with one another. For this reason, synergy should be interpreted cautiously and in a context-dependent manner rather than assumed *a priori* for all combined interventions. The shared molecular basis underlying these interactions is summarized in **Figure 2**.

**Figure 2:**
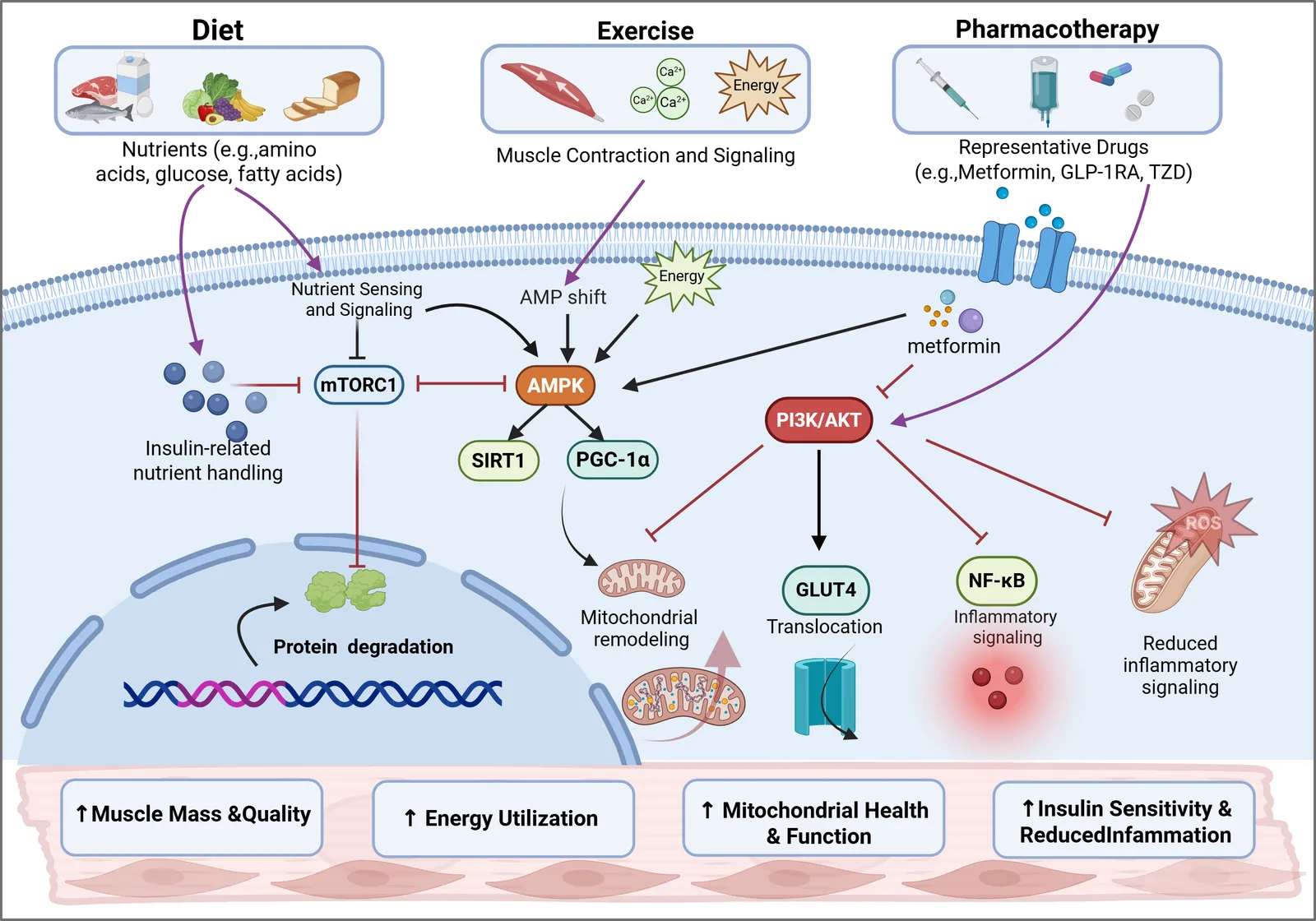
Convergent molecular pathways through which diet, exercise, and pharmacotherapy regulate skeletal muscle metabolism. Dietary signals, exercise-induced contraction, and pharmacological interventions influence skeletal muscle through partially overlapping intracellular pathways rather than fully independent mechanisms. Nutrient availability and amino acid sensing promote anabolic signaling through mTORC1, whereas exercise-derived energetic stress activates AMPK and downstream mitochondrial regulators, including SIRT1 and PGC-1α. Pharmacological inputs, represented here by agents such as metformin and insulin-sensitizing drugs, intersect with AMPK- and PI3K/AKT-related pathways to modulate glucose handling, inflammatory signaling, and mitochondrial remodeling. These shared molecular networks collectively regulate protein turnover, energy utilization, mitochondrial quality, insulin sensitivity, and inflammatory tone, thereby linking multimodal interventions to improvements in skeletal muscle quality and metabolic homeostasis. Created in BioRender. Liu, X. (2026). https://BioRender.com/j0y90ep.

The best-supported example of a beneficial interaction remains the combination of protein intake and resistance exercise. Protein supplementation can augment resistance training-induced gains in lean mass and strength, although the magnitude of benefit varies with age, habitual protein intake, and training status (Morton et al., 2018). Under conditions of energy restriction, higher protein intake combined with intense exercise also helps preserve lean mass and promotes a more favorable body-composition response (Longland et al., 2016). Taken together, current evidence indicates that anabolic nutrition and resistance exercise converge on common regulatory mechanisms in skeletal muscle, making this one of the few areas in which the clinical relevance of a combined intervention is supported by relatively strong human evidence.

A second important area concerns metabolic flexibility under nutrient excess or metabolic dysfunction. Skeletal muscle in obesity or during high-fat feeding often shows an impaired ability to adjust fatty acid oxidation appropriately, and exercise training can partially restore this adaptive response (Battaglia et al., 2012; Fritzen et al., 2020). At the same time, the quality of the dietary signal remains important. A high-phosphate diet, for example, can impair exercise tolerance and disrupt fatty acid metabolism, indicating that not all nutritional environments support adaptive remodeling in muscle (Peri-Okonny et al., 2019). These observations suggest that the value of combined lifestyle intervention lies not simply in increasing the number of inputs, but in aligning diet and exercise toward the same metabolic program.

This point is especially relevant for antioxidant or redox-active nutritional support. Although such interventions may reduce some indices of oxidative stress, ROS also participate in the signaling processes that underlie mitochondrial adaptation and training responsiveness. N-acetylcysteine supplementation has been reported to alter skeletal muscle signaling and attenuate inflammatory and recovery-related responses after intense eccentric exercise (Michailidis et al., 2013). More broadly, human studies have shown that antioxidant supplementation can blunt some of the health-promoting and cellular adaptations normally induced by exercise training (Ristow et al., 2009; Paulsen et al., 2014). Thus, combined interventions should not be judged solely by their ability to suppress inflammation or oxidative stress, but by whether they preserve the signaling environment required for functional adaptation. At the whole-diet level, the Mediterranean diet represents a broader antioxidant and anti-inflammatory approach, with emerging evidence suggesting potential benefits for antioxidant status and muscle-related outcomes (Bianchi et al., 2024).

The interaction between pharmacotherapy and exercise is similarly nuanced. It is often assumed that drugs targeting metabolic pathways will necessarily enhance the benefits of exercise, but available evidence does not support such a general conclusion. In individuals with prediabetes, the combination of exercise training and metformin did not consistently improve insulin sensitivity beyond exercise alone (Malin et al., 2012). In older adults, metformin has also been shown to attenuate mitochondrial adaptations to aerobic exercise training (Konopka et al., 2019). These findings are clinically important because they suggest that pharmacological treatment may, in some settings, modify or even constrain exercise-induced remodeling in skeletal muscle.

GLP-1 receptor agonist-induced weight loss represents another clinically relevant setting in which adequate protein intake and resistance exercise may help preserve muscle mass and function, although direct evidence for these combined strategies remains limited (Simsek and Ucar, 2026).

At the same time, selected pharmacological or nutritional adjuncts may still be useful in settings where muscle quality or adaptive capacity is already compromised. In preclinical models, maintenance of LAT1 activity and branched-chain amino acid support can counter muscle atrophy, preserve mitochondrial content, or improve muscle status during chemotherapy, detraining, or muscle disease (Banfi et al., 2018; Matsunaga et al., 2022; Mora and Adegoke, 2024). Other compounds, including narciclasine, pioglitazone, and trimetazidine, have shown the ability to improve oxidative metabolism, mitochondrial quality control, or exercise capacity in animal models of diet-induced or diabetic muscle dysfunction (Takada et al., 2014; Julien et al., 2017; Zhang et al., 2021). Similarly, green tea catechins combined with exercise have shown beneficial effects on mitochondrial capacity and satellite cell-related responses in aged muscle (Matsuzaki et al., 2025). However, these findings remain weighted toward animal or mechanistic studies, and should not yet be interpreted as established clinical combination strategies.

Overall, skeletal muscle should be viewed as an integration site where nutritional, mechanical, and pharmacological signals are filtered rather than simply accumulated. The strongest human evidence currently supports protein-based nutritional support combined with resistance training, particularly for preserving or increasing lean mass. Other combinations are better regarded as mechanism-based opportunities rather than established synergistic therapies. Future work should therefore move beyond broad claims of synergy and instead identify which patients are most likely to benefit, under which metabolic conditions, and through which shared regulatory pathways in skeletal muscle. The translational implications of these multimodal interactions can be organized according to clinical context, evidence maturity, and precision determinants, as summarized in **Table 1**; **Figure 3**.

**Table 1:** Representative modes of mechanistic convergence among diet, exercise, and pharmacotherapy in skeletal muscle.

Intervention combination	Shared skeletal muscle node(s)	Principal outcome(s)	Evidence level	Key limitation
Dietary protein + resistance exercise training (Morton et al., 2018)	Amino acid sensing, mTORC1 activation	Increased muscle protein synthesis, lean mass, and strength	Strong human evidence	The most robust example of beneficial convergence, although formal synergy not directly tested
Higher-protein diet + resistance exercise during energy restriction (Longland et al., 2016)	Protein turnover control, preservation of anabolic signaling under energy deficit	Attenuated muscle loss and improved protein homeostasis	Moderate human evidence	Effects are influenced by the severity of energy deficit, training status, and age
Whey-plus-collagen supplementation + exercise recovery (Aussieker et al., 2025)	Myofibrillar and connective tissue protein synthesis	Broader structural support for post-exercise recovery	Early human evidence	Evidence remains limited, and the long-term functional superiority over standard protein strategies is not yet established
Endurance-type exercise + favorable dietary lipid composition/bioactive nutrients (Battaglia et al., 2012; Peri-Okonny et al., 2019; Fritzen et al., 2020)	AMPK-SIRT1-PGC-1α signaling, fatty acid handling, mitochondrial remodeling	Improved lipid oxidation, mitochondrial function, and metabolic flexibility	Moderate preclinical evidence with limited human support	Strongly context-dependent and less established than protein-resistance exercise interactions
Exercise + dietary nitrate or cocoa flavanols (Pawlak-Chaouch et al., 2016; Decroix et al., 2018)	Microvascular support, redox regulation, glucose metabolism-related signaling	Improved metabolic efficiency and attenuation of inflammatory disturbances	Emerging evidence	Findings are heterogeneous and depend on dose, training background, and metabolic status
Exercise + N-acetylcysteine supplementation (Ristow et al., 2009; Michailidis et al., 2013; Paulsen et al., 2014)	Redox-sensitive signaling pathways	Reduced oxidative stress and inflammatory responses	Mixed evidence	May blunt adaptive signaling required for normal training-induced remodeling; not necessarily synergistic
Branched-chain amino acids or selective amino acid support + recovery/rehabilitation states (Banfi et al., 2018; Matsunaga et al., 2022; Mora and Adegoke, 2024)	Amino acid availability, mitochondrial maintenance, recovery-related metabolic resilience	Preservation of mitochondrial content and attenuation of functional decline	Preclinical to early translational evidence	More relevant in catabolic or detraining settings than in healthy adaptation; clinical generalizability remains limited
GLP-1 receptor agonism + muscle-preserving adjunct strategies (Nunn et al., 2024; Pantazopoulos et al., 2025; Simsek and Ucar, 2026)	Body-composition remodeling, preservation of muscle metabolic function during weight loss	Fat loss with partial protection of lean mass	Emerging preclinical and translational evidence	Promising concept, but the optimal combination framework for clinical muscle preservation remains undefined
Metabolic agents (e.g., pioglitazone, trimetazidine, narciclasine) + lifestyle intervention (Takada et al., 2014; Julien et al., 2017; Zhang et al., 2021)	Oxidative metabolism, mitochondrial function, insulin sensitivity	Improved exercise capacity and metabolic adaptation in compromised muscle	Predominantly preclinical evidence	Clinical interaction models remain insufficiently defined, and effects may not be generalizable across disease contexts

AMPK, AMP-activated protein kinase; PGC-1α, peroxisome proliferator-activated receptor gamma coactivator 1-alpha; SIRT1, sirtuin 1; GLP-1, glucagon-like peptide-1.

Evidence level reflects the overall maturity of the literature rather than formal quantitative proof of greater-than-additive interaction. In several settings, combined interventions may be additive, neutral, or context-dependently antagonistic rather than truly synergistic.

**Figure 3:**
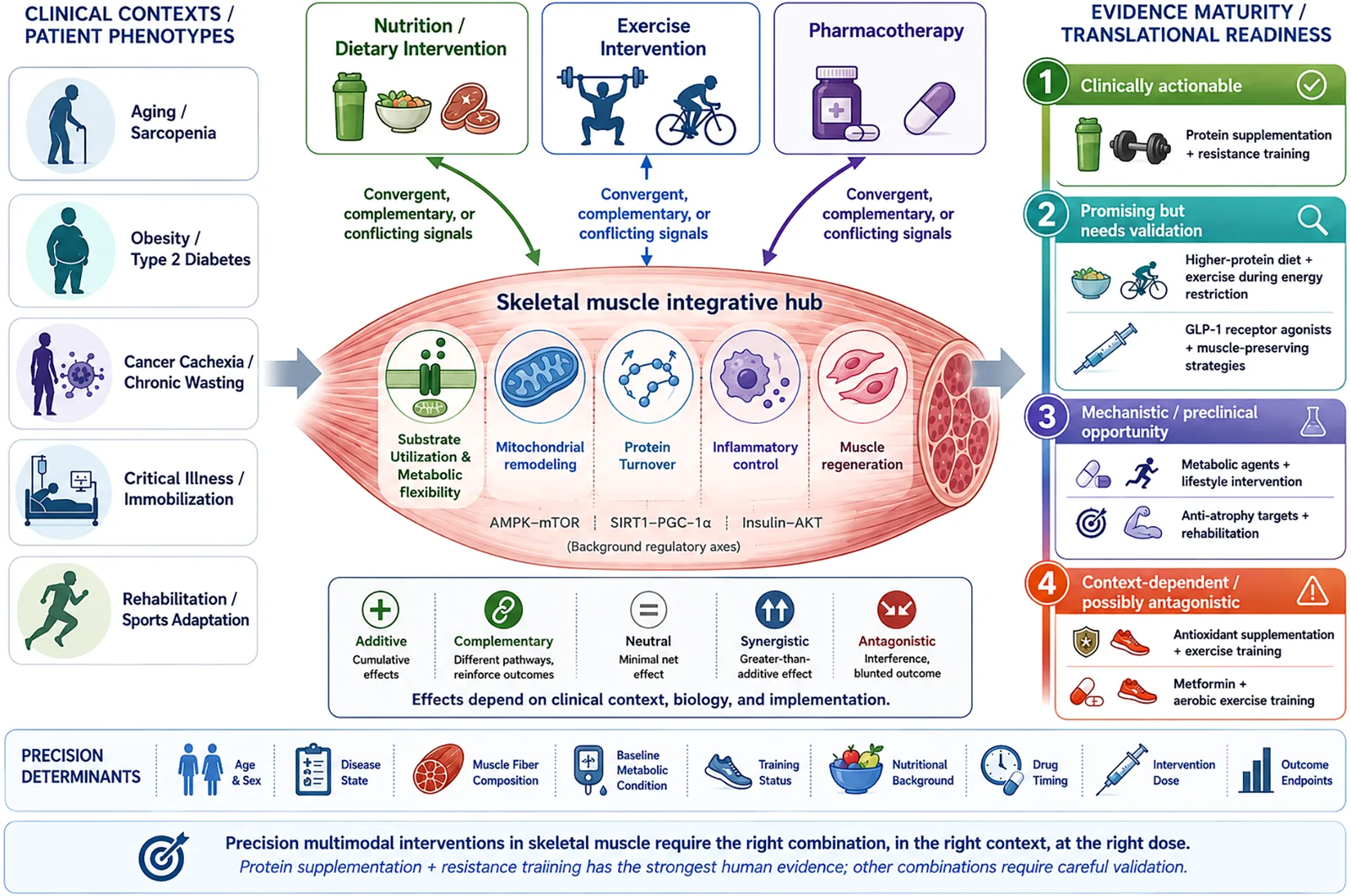
Evidence-to-translation framework for precision multimodal interventions targeting skeletal muscle metabolism. This framework organizes combined nutritional, exercise-based, and pharmacological interventions according to clinical context, evidence maturity, and precision determinants. Skeletal muscle functions as an integrative hub for multimodal signals, but combined effects may be additive, complementary, neutral, synergistic, or antagonistic. Protein supplementation plus resistance training currently has the strongest human evidence, whereas other combinations require context-specific validation.

## 6 Conclusions and outlook

As the body’s largest metabolic organ, skeletal muscle plays a pivotal role in maintaining energy homeostasis, regulating insulin sensitivity, and supporting systemic metabolic health. Existing research consistently demonstrates that dietary, exercise, and pharmacological interventions do not act in isolation on skeletal muscle. Instead, they form a highly coupled metabolic regulatory system through multi-level networks involving energy sensing, nutrient signaling, mitochondrial homeostasis, inflammatory regulation, and myogenic factors. The convergence of diverse interventions at the skeletal muscle level determines muscle efficiency in utilizing glucose, lipids, and amino acids, thereby influencing systemic metabolic phenotypes. Therefore, shifting from a “single-factor regulation” to an “integrated multi-intervention mechanism” research paradigm is crucial for understanding skeletal muscle metabolic plasticity.

At the mechanistic level, skeletal muscle exhibits significant context-dependent responses to nutrient supply, mechanical stimuli, and drug signals. Exercise reshapes skeletal muscle sensitivity to nutrients and drugs, amplifying anabolic processes and mitochondrial adaptation. Dietary composition not only supplies metabolic substrates but also modulates muscle signaling pathways through amino acid sensing, lipid signaling, and metabolic intermediates. Drug interventions, meanwhile, finely regulate key metabolic nodes within specific pathological contexts. The cross-talk among these three components across AMPK-mTOR, SIRT-PGC-1α, insulin/AKT, and inflammation-related pathways forms the core network of skeletal muscle metabolic regulation. This framework also provides a unified mechanism for explaining the effects observed in multimodal interventions.

Looking ahead, skeletal muscle metabolism research urgently needs to transition from descriptive correlations to mechanism-driven precision interventions. First, we should systematically decipher the heterogeneous basis of interactive effects among diet, exercise, and drugs across different populations, disease states, and muscle fiber types. Second, multi-omics integration and temporal analysis will help reveal key metabolic nodes and signaling “windows” during combined interventions, providing evidence for optimizing intervention timing and dosage. Additionally, future multimodal studies should consider whole dietary patterns, rather than isolated nutrients alone, when evaluating nutritional interactions with exercise and pharmacotherapy. Furthermore, as an endocrine organ, skeletal muscle’s secretory factors play an under-explored role in cross-organ metabolic regulation, potentially serving as crucial mediators linking nutrition, exercise, and drug effects. Overall, establishing multidimensional metabolic intervention strategies centered on skeletal muscle will provide a more forward-looking and translationally valuable theoretical foundation for preventing metabolic diseases, promoting healthy aging, and advancing sports medicine.
